# Dual-Band MIMO Antenna with Enhanced Isolation for 5G NR Application

**DOI:** 10.3390/mi14010095

**Published:** 2022-12-30

**Authors:** Shuqi Xi, Jing Cai, Lingrong Shen, Qiangjuan Li, Gui Liu

**Affiliations:** College of Electrical and Electronic Engineering, Wenzhou University, Wenzhou 325035, China

**Keywords:** dual-band, 5G NR, high isolation, MIMO antenna

## Abstract

A two-port multiple-input and multiple-output (MIMO) antenna with dual-band characteristics operating at the fifth-generation (5G) new radio (NR) sub-6 GHz n7/n38/n41/n79 bands is proposed. The proposed MIMO antenna is composed of two symmetric antenna elements and a defected ground plane. The antenna element consists of an incomplete circular patch with two L-shaped branches. By applying the defected ground structure and the slotted stub, the current distribution on the ground plane is changed to reduce the mutual coupling between the antenna elements. The measured −10 dB reflection coefficients cover 2.34–2.71 GHz and 3.72–5.10 GHz, while the measured isolation is larger than 20 dB at the whole operating frequency band. The paper has investigated different performance parameters in terms of the envelope correction coefficient (ECC), diversity gain (DG), radiation patterns, antenna gain, and efficiency. The proposed MIMO antenna is suitable for 5G applications.

## 1. Introduction

Nowadays, the fifth generation (5G) communication system, when it is compared with traditional communication systems, has the advantages of high speed, wide frequency bandwidth, low power consumption, and high reliability. Meanwhile, the multiple-input and multiple-output (MIMO) antenna system has attracted extensive attention due to it having lower multipath fading effects, higher channel capacity, and an increased transmission rate than those of the single antenna communication system. However, there are still many challenges in designing high-isolation and compact-size MIMO antennas, such as the mutual coupling between the antenna elements [1].

Therefore, it is a critical problem in the design process of the MIMO antenna to reduce mutual coupling. Many scholars have presented various decoupling techniques to reduce mutual coupling between antennas [2,3,4,5,6,7]. In [2], an ultrawideband (UWB) MIMO antenna achieved high isolation values that are larger than 20 dB for the whole operating band by slotted stubs. A coplanar waveguide-fed MIMO antenna with high isolation values was proposed by adding a double Y-shaped branch [3]. In [4], by introducing a creative un-protruded multi-slot (UPMS) isolating element, a four-port MIMO antenna array was proposed to reduce the mutual coupling between the antenna elements further. In [5], each antenna element was placed orthogonally to achieve the good isolation of 45 dB at the band of 36.83–40.0 GHz. It is reported in [6] that a combination of a defected ground structure (DGS) and electromagnetic band gaps (EBG) can be adopted to ease mutual coupling. In [8], the mutual coupling of a four-port MIMO antenna has been reduced by the orthogonal orientation of radiating elements rather than any decoupling structures.

Furthermore, it is also challenging to design a multi-band MIMO antenna to meet substantial wireless communication bands because the same isolation method may not work at different frequency bands. Recently, several dual-band MIMO antennas have been proposed [9,10,11,12,13,14,15,16,17,18,19,20]. In [9], a dual-notched four-element MIMO antenna with dual-band characteristics has been proposed by introducing gap sleeves and an H slot. It covers the frequency bands of 3.3–4.1 GHz and 8.2–8.6 GHz. A dual-band MIMO antenna was proposed in [10] by implementing an L-shaped feeding strip, a parasitic rectangle strip, and a modified Z-shaped radiating strip. In [13], an eight-element MIMO antenna was designed to meet the requirement of the 5G mobile terminals. A quad-element MIMO antenna with E-shaped and G-shaped stubs was presented to achieve two resonances at 1.5 GHz and 2.45 GHz, respectively in [18]. In [20], a MIMO antenna array was realized which can cover the 5G NR Bands n77/n78/n79 and WLAN 5 GHz band.

In this paper, a two-port MIMO antenna with high isolation and dual-band characteristics is presented. The antenna consists of two identical three-quarter circular patches which are placed symmetrically on the top of the substrate, with the size of 41 × 30 × 1.59 mm^3^. The antenna generates two frequency resonances by introducing two L-shaped branches and two step-shaped gaps in the radiators. The working bands can cover n7 (2.5–2.69 GHz), n38 (2.57–2.62 GHz), n41 (2.496–2.690 GHz), and n79 bands (4.4–5 GHz). The measured isolation is larger than 20 dB. Other MIMO antenna parameters are presented to analyze the performance, such as the S-parameters (reflection and transmission coefficients), radiation patterns, ECC, DG, and efficiencies.

## 2. Antenna Design and Analysis

### 2.1. Antenna Geometry

**Figure 1:**
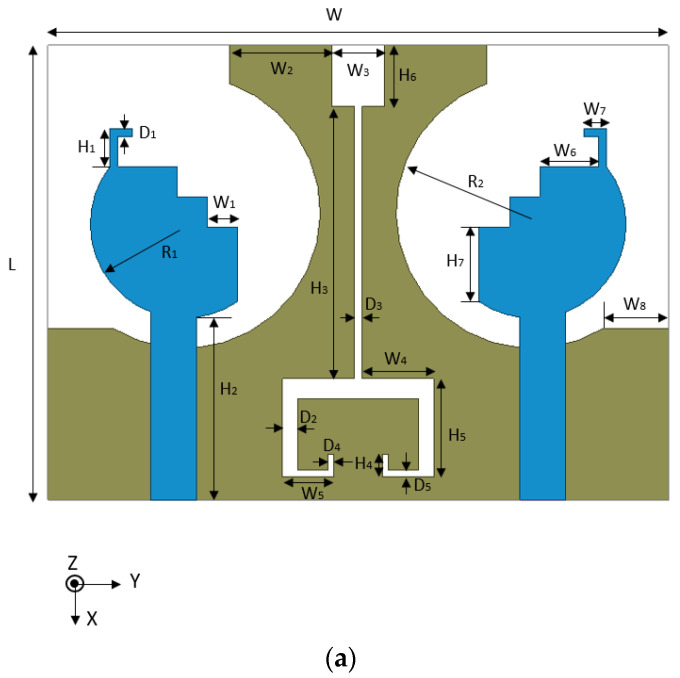

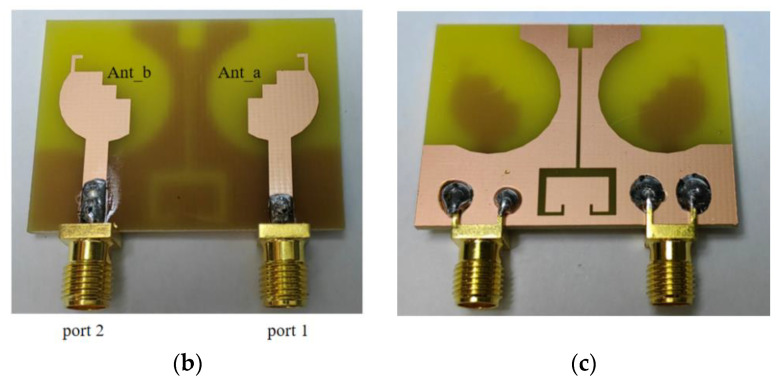
The geometry and prototype of the proposed design: (**a**) perspective view, (**b**) top view, and (**c**) bottom view.

The configuration and prototype of the designed dual-band antenna are shown in Figure 1. The radiator of the antenna element consists of an L-shaped strip and a defective circular radiator composed of a semicircle with step-shaped strips. The radiator is fed by a 50 Ω micro-strip feedline. Two identical antenna elements are printed on a 1.59 mm thick FR4 substrate with relative permittivity of 4.4 and loss tangent of 0.02. A cambered ground plane is printed on the bottom of the substrate. High isolation is achieved by etching a C-shaped slot, a rectangular slot, and a rectangular slit. Through simulation and optimization, the detailed parameters are shown in Table 1.

### 2.2. Design Process

**Figure 2:**
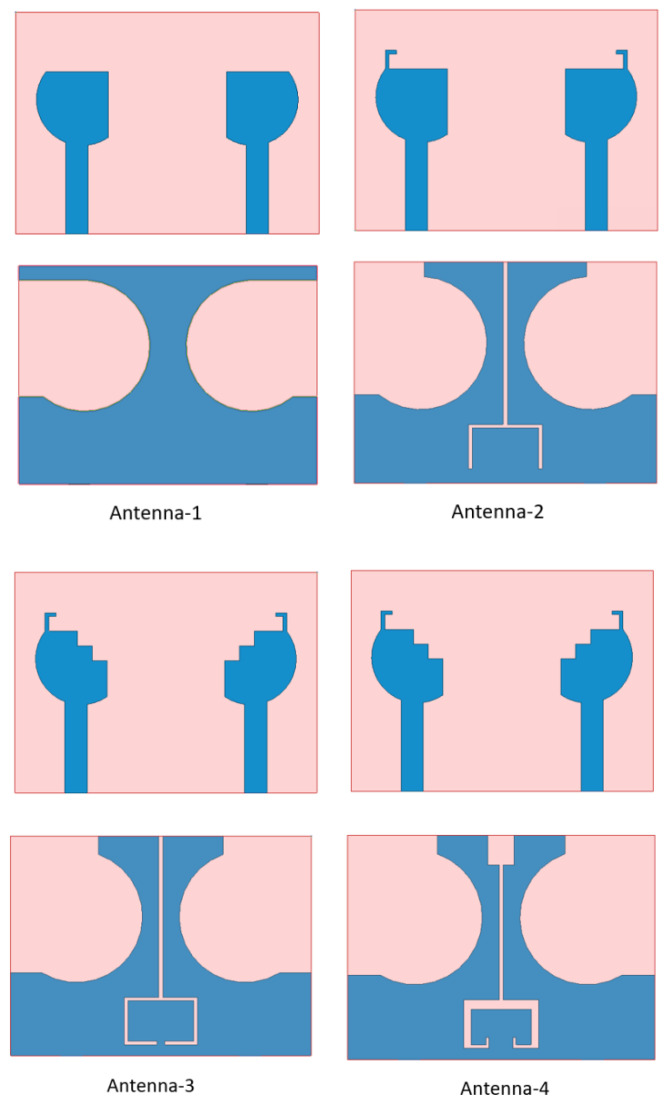
Design stages of the proposed design.

**Figure 3:**
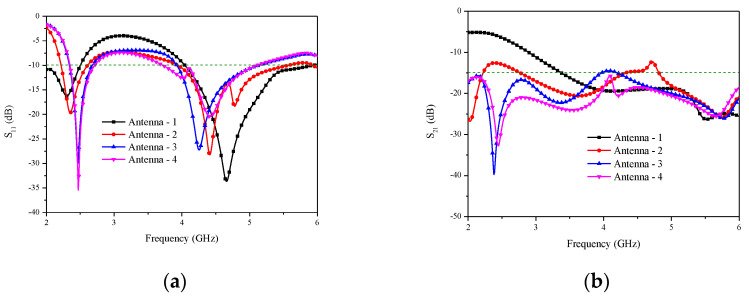
Design stage responses of the intended two-port MIMO antenna: (**a**) S_11_ responses and (**b**) S_21_ responses.

Figure 2 presents the detailed process for the proposed antenna to study the performance of the 5G dual-band monopole antenna. Because two antenna elements are placed symmetrically to share the ground plane, S_11_ and S_21_ are the same as S_22_ and S_12_, respectively. For simplicity, we only discuss S_11_ and S_21_. The simulated S-parameters are plotted in Figure 3. In Antenna 1, a three-quarters circular radiation patch with the circular slotted ground plane is designed. Antenna 1 can achieve a −10 dB impedance bandwidth of 500 MHz (2–2.5 GHz) and 1880 MHz (4.05–5.93 GHz). However, the isolation between the radiating elements is non-ideal over the operation bands, which is illustrated in Figure 3b. In Antenna 2, an L-shaped branch is designed to realize a lower frequency shift (right) and a higher frequency shift (left), which can be seen in Figure 3a. By adding an inverted U-shaped slot and an I-shaped slot on the back side, it can reduce the mutual coupling between the antenna elements. In addition, as shown in Antenna 3, introducing the step-shaped radiating elements can realize useful impedance bandwidth. The result indicates that the impedance bandwidth covers 2.33–2.68 GHz and 3.93–5.13 GHz, and the value of S_21_ adjacent to the defected ground structure decreases to below −24 dB and −17 dB in the 2.5 GHz and 4.5 GHz bands, respectively. Finally, a T-shaped slot and a G-shaped slot are incorporated at the bottom plane to reduce the mutual coupling to below −15 dB for the entire frequency band.

### 2.3. Parametric Study

**Figure 4:**
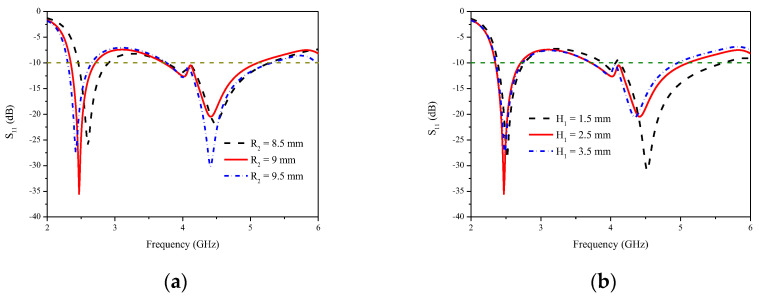
Reflection coefficients of the proposed MIMO antenna (**a**) with different values of R_2_ and (**b**) with different values of H_1_.

The radiators can generate two resonances. The incomplete circular radiation patch generates the lower resonance of the antenna, and the L-shaped antenna branches can generate the higher resonance. Figure 4 describes the two resonance frequencies, which can be independently tuned by changing the values of R_2_ and H_1_. When the value of R_2_ increases, the lower resonance shifts to the lower frequencies, as is shown in Figure 4a. When the value of H_1_ increases, the ground current path increases, the lower resonance frequency remains at 2.5 GHz, the higher resonance frequency shifts to the lower frequencies, and the −10 dB bandwidth of the higher frequency band is reduced. The optimum values of R_2_ and H_1_ are 9 mm and 2.5 mm, respectively.

### 2.4. Current Distribution

**Figure 5:**
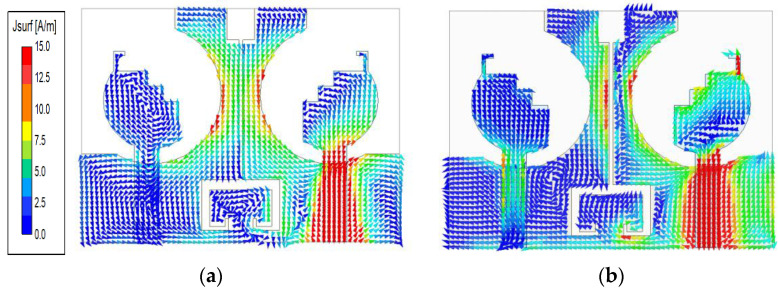
Current distributions of the proposed MIMO antenna system at (**a**) 2.5 GHz and (**b**) 4.7 GHz.

Figure 5 shows the simulated current distributions of the proposed antenna at 2.5 GHz and 4.7 GHz. After adding the DGS, one can see that the current from port 1 to port 2 is cut off. Therefore, high isolation is achieved because the surface current and near fields are concentrated within the decoupling structure. 

When Ant_a is excited, the current is mainly concentrated on the edge of a circular patch at 2.5 GHz. While when it is working at 4.7 GHz, there is a strong current on the L-shaped branch and step-shaped part. Thus, each branch in the radiation elements is responsible for stimulating resonance. The formula controlling the relationship between excitation resonant frequency, antenna geometric parameters, and the physical characteristics is as follows:

(1) $$f_{r}=\frac{c}{2L_{e}}\sqrt{\frac{2}{\epsilon_{r}+1}}$$

where *Le* is the total electric length of the structural antenna elements.

## 3. Results and Discussion

### 3.1. S-Parameters

**Figure 6:**
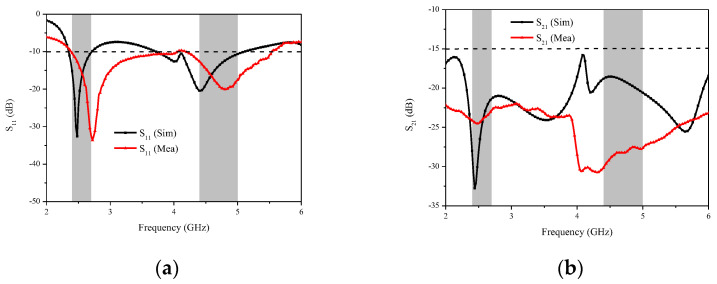
Simulated and measured S-parameters of the proposed MIMO antenna system: (**a**) S_11_ and (**b**) S_21_.

Figure 6 presents the simulated and measured S-parameters results for port 1, including S_11_ and S_21_. It is exhibited that the measurement results are different from the simulation results because of SMA soldering and unavoidable tolerances in the fabrication and measurement process. To be specific, it is observed that the measured −10 dB impedance band can cover the n7 (2.5–2.69 GHz), n38 (2.57–2.62 GHz), n41 (2.496–2.690 GHz), and n79 bands (4.4–5 GHz) in Figure 6a. From the transmission curves, it can be seen that S_21_ is below −20 dB over the whole operating frequency band (2.0–6.0 GHz).

### 3.2. Radiation Pattern

**Figure 7:**
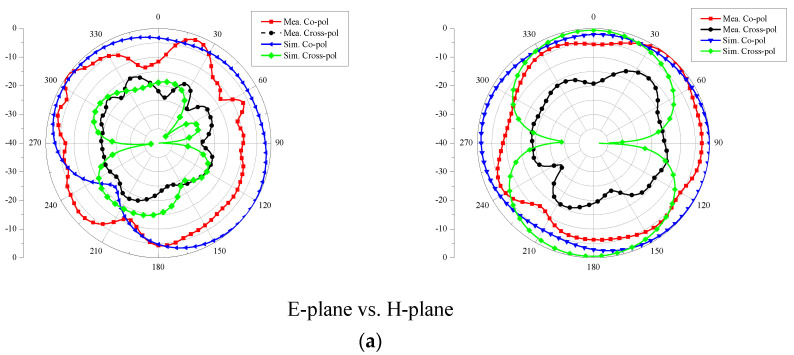

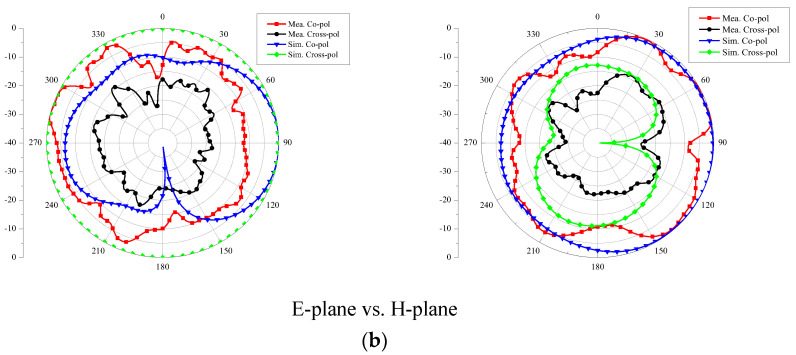
Two-dimensional radiation patterns at E-plane and H-plane for the intended two-port MIMO antenna at port-1: (**a**) 2.5 GHz and (**b**) 5.7 GHz.

To study the radiation mechanism of the antenna in-depth, Figure 7 presents the measured far-field radiation patterns in the H-plane and the E-plane at 2.5 GHz and 4.7 GHz, respectively. During the measurement and simulation, one port is excited, while the other one is matched with a 50 Ω load terminal. The proposed antenna has nearly stable omnidirectional radiation patterns in the H-plane at 2.5 GHz and 4.7 GHz. At 2.5 GHz, the E-plane is shown as a bidirectional model, while the radiation patterns appear deformed in the E-plane at 4.7 GHz. There is no effect on the radiation performance.

## 4. The Performance Evaluation of MIMO Antenna Systems

### 4.1. The Realized Peak Gain and Efficiency

**Figure 8:**
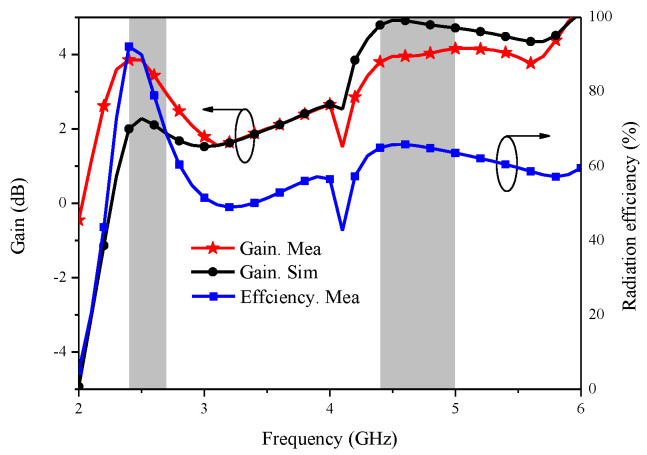
Gain and radiation efficiency of the proposed MIMO antenna.

Gain, as one of the critical parameters of the antenna, is used to measure the antenna’s ability to receive and transmit signals. Figure 8 shows the gain and radiation efficiency of the proposed MIMO antenna. It is presented that the peak gain of the proposed antenna is around 3 dBi for the 2.5 GHz band, and it is around 3.8 dBi for the 4.7 GHz band, while the measured radiation efficiency is higher than 62% and 66% in the operating bands, respectively.

### 4.2. Envelope Correlation Coefficient and Diversity Gain

In addition, gain, the envelope correlation coefficient, and diversity gain are also considered to be valuable parameters to evaluate the antenna system performance. ECC represents the degree of correlation between the antenna elements of a multiple antenna system. The calculation formulas of ECC calculated from the S-parameters or far-field radiation characteristics are shown in Equations (2) and (3) [16], respectively. However, only when the antenna efficiencies are nearly 100%, is Equation (2) accurate. Therefore, ECC is calculated using Equation (3) in this paper.

(2) $$\mathrm{ECC}=\frac{{|S}_{11}^{*}S_{12}{+S}_{22}^{*}S_{21}{|}^{2}}{(1\;-{\left|S_{11}\right|}^{2}{+|S}_{21}{|}^{2})(1\;-{\left|S_{22}\right|}^{2}{+|S}_{12}{|}^{2})}$$

(3) $$\rho_{e}=\iint \frac{[\vec{F_{1}}(\theta ,\varphi )\times \vec{F_{2}}(\theta ,\varphi )]d\partial^{2}}{\iint {\left|\vec{F_{1}}\left(\theta ,\varphi \right)\right|}^{2}d\partial \iint {\left|\vec{F_{2}}\left(\theta ,\varphi \right)\right|}^{2}d\partial }$$

where ‘F_i_(θ,φ)’ is the radiated field of the first antenna.

On the other hand, the DG acts as the other index to evaluate the MIMO antenna isolation performance. The diversity gain is calculated through the following Equation (4):

(4) $$\mathrm{DG}=10\sqrt{1\;-{\left(\mathrm{ECC}\right)}^{2}}$$

**Figure 9:**
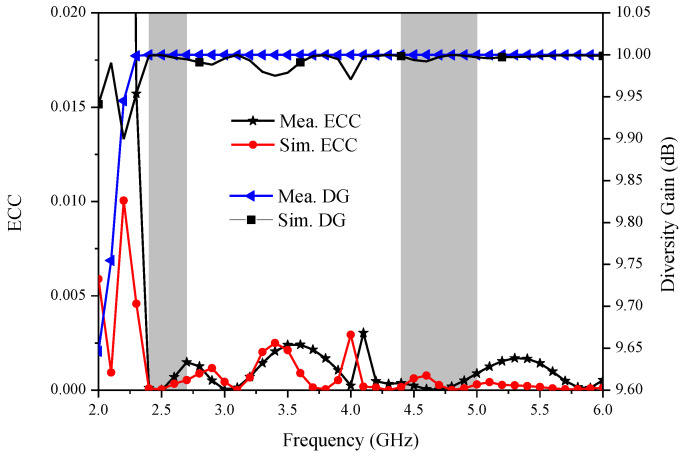
Measured and simulated ECC and DG of the proposed two-element MIMO antenna.

Figure 9 depicts the measured values and simulated values of ECC and DG for the proposed MIMO antenna, respectively. It can be seen that the ECC is below 0.005, and the DG is greater than 9.65 dBi within the operating bands, which meets the engineering standard. A performance comparison of the proposed antenna with previous dual-band antennas is provided in Table 2. In this table, it can be observed that the presented dual-band MIMO antenna has achieved a more compact size, a lower ECC, and a higher isolation compared to those of the other referenced antennas.

## 5. Conclusions

In this paper, a dual-band MIMO antenna with high isolation has been designed. The size of the proposed antenna is 41 × 30 × 1.59 mm^3,^ and the impedance matching (which is better than −10 dB) covers 5G NR sub-6 GHz n7/n38/n41/n79. The high isolation of a value that is better than 20 dB is successfully realized by adding the defected ground structure and the slotted stub between the two antenna elements. Moreover, it is found that the antenna’s lower gain and efficiency for the proposed system are 3 dBi and 62%, respectively. The measured ECC is less than 0.005, which meets the requirement of the MIMO systems. Therefore, the experimental results and the designed MIMO antenna structure exhibit that the presented antenna is preferred for 5G communication applications.

## Figures and Tables

**Table 1:** Parameters of the presented antenna (unit: mm).

Parameter	W_1_	W_2_	W_3_	W_4_	W_5_	W_6_
Value	2	6.75	3.5	4.75	3.4	3.9
Parameter	W_7_	W_8_	H_1_	H_2_	H_3_	H_4_
Value	1.5	4.34	2.5	12.05	18	1.5
Parameter	H_5_	H_6_	D_1_	D_2_	D_3_	D_4_
Value	6.5	4	0.5	1	0.5	0.4
Parameter	D_5_	R_1_	R_2_	W	L	
Value	0.5	6.2	9	41	30	

**Table 2:** Performance comparison with dual-band antennas.

Ref.	Operating Bands (GHz)	Isolation (dB)	ECC	Gain (dBi)	Size (mm^3^)
[7]	2.25–2.9 5.05–6.025	>19.3	<0.03	2.4 and 3.8	50 × 50 × 1.6
[12]	2.23–2.46 3.22–4.04	>12	10^−5^ and 0.002	3.6 and 7.1	105 × 105 × 1.83
[13]	2.5–2.7 4.8–5.0	17.96 and 20.1	0.006 and 0.12	4.35 and 4.6	38.5 × 38.5 × 1.59
[15]	2.4–2.5 5.1–5.8	15	<0.2	1.5 and 1.5	38 × 38 × 1.6
[16]	2.3–2.5 5–5.2	20 and 20	<0.05	1.28 and 2.1	38 × 42 × 0.8
[17]	2.25–2.41 4.7–6.25	>18	<0.2	1.7 and 3	48 × 48 × 1.6
This work	2.34–2.71 3.72–5.10	21 and 18	<0.005 and <0.001	3 and 3.8	41 × 30 × 1.59

## Data Availability

Data are contained within the article.
